# Treating primary immunodeficiencies with defects in NK cells: from stem cell therapy to gene editing

**DOI:** 10.1186/s13287-020-01964-5

**Published:** 2020-10-27

**Authors:** C. Eguizabal, L. Herrera, M. Inglés-Ferrándiz, J. C. Izpisua Belmonte

**Affiliations:** 1 Cell Therapy, Stem Cells and Tissues Group, Biocruces Bizkaia Health Research Institute, Barakaldo, Spain; 2 Research Unit, Basque Center for Blood Transfusion and Human Tissues, Osakidetza, Galdakao, Spain; 3 Gene Expression Laboratory, The Salk Institute for Biological Studies, 10010 North Torrey Pines Road, La Jolla, CA 93027 USA

**Keywords:** Primary immunodeficiency diseases (PIDs), Induced pluripotent stem cells (iPSCs), Gene editing, CRISPR-Cas9, Stem cell therapy, Hematopoietic stem cells, Natural killer cells

## Abstract

Primary immunodeficiency diseases (PIDs) are rare diseases that are characterized by genetic mutations that damage immunological function, defense, or both. Some of these rare diseases are caused by aberrations in the normal development of natural killer cells (NKs) or affect their lytic synapse. The pathogenesis of these types of diseases as well as the processes underlying target recognition by human NK cells is not well understood. Utilizing induced pluripotent stem cells (iPSCs) will aid in the study of human disorders, especially in the PIDs with defects in NK cells for PID disease modeling. This, together with genome editing technology, makes it possible for us to facilitate the discovery of future therapeutics and/or cell therapy treatments for these patients, because, to date, the only curative treatment available in the most severe cases is hematopoietic stem cell transplantation (HSCT). Recent progress in gene editing technology using CRISPR/Cas9 has significantly increased our capability to precisely modify target sites in the human genome. Among the many tools available for us to study human PIDs, disease- and patient-specific iPSCs together with gene editing offer unique and exceptional methodologies to gain deeper and more thorough understanding of these diseases as well as develop possible alternative treatment strategies. In this review, we will discuss some immunodeficiency disorders affecting NK cell function, such as classical NK deficiencies (CNKD), functional NK deficiencies (FNKD), and PIDs with involving NK cells as well as strategies to model and correct these diseases for further study and possible avenues for future therapies.

## Background

Primary immunodeficiency disorders (PIDs) are rare diseases caused by genetic mutations that damage immunological function, defense, or both. PIDs refer to over 130 disorders that result from developmental and/or functional defects in one or more cell types of the immune system. PIDs are generally classified as disorders of adaptive immunity (B cell, T cell, or combined immunodeficiencies) or innate immunity (NK cells, phagocyte, and complement disorders). Some of these diseases affect natural killer (NK) cells [1, 2].

NK cells are lymphocytes of the innate immune system poised to deliver a response immediately after recognizing specific signals of “danger,” stress, or foreign origin. NK cells are initially defined as rapid cell-mediated cytotoxicity cells, though they also initiate a slower receptor-mediated apoptosis, and efficiently produce soluble mediators, such as cytokines, and provide contact-dependent co-stimulation. Hence, NK cells contribute to the regulation of immune responses, in the surveillance of stress and cancer cells, and in the defense against infections [3]. While these functions are not exclusive to NK cells, the ability to quickly mediate effector functions without the need of further development and/or maturation is a crucial distinctive feature of mature NK cells and cytotoxic T lymphocytes (CTLs), both of which are efficient at mediating cytotoxicity.

NK cell effector functions happen after ligation of germline-encoded receptors and involve the secretion of cytolytic molecules. These molecules are contained in pre-formed lytic granules of resting human NK cells. As a result, the cytolytic process needs to be well controlled and may involve additional or enhanced mechanisms for controlling the secretion of lytic granule contents [4].

Several NK cell deficiencies have been identified as being present in PIDs, including some characterized by an impediment in their development (classic NK deficiencies or CNKD) or by an aberrant formation of the lytic synapse (functional NK deficiencies or FNKD). To date, only one FNKD has been shown to impair only NK cell function. Many studies on how the lytic synapse is formed have been performed with T cells from patients with PIDs. Though cytotoxic lymphocytes have critical roles in host defense and immune regulation, NK cell deficiency can add to the clinical phenotypes [5].

**Fig. 1:**
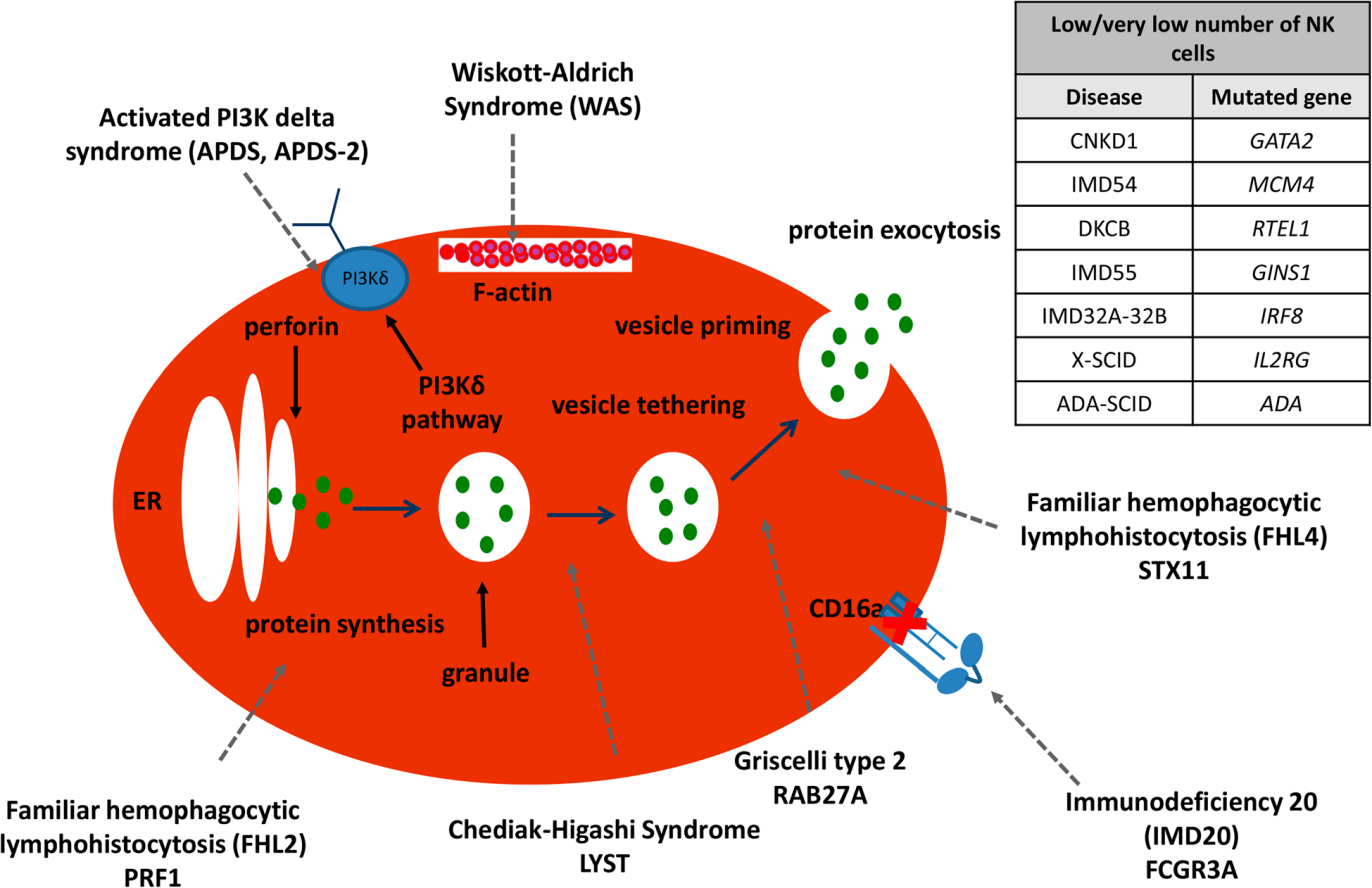
Functional and cell number defects in NK cells caused by different PIDs. Different check points of cytotoxicity may be affected in some diseases, such as FHL2, FHL4, CHS, and GS2. Other diseases may affect the proper functioning of a signaling pathway (APDS, APDS-2, IMD20) or the correct assembly of the cytoskeleton (WAS). Diseases with cell number defects in NK cells, such as CNKD1, IMD54, DKCB, IMD55, IMD32A-32B, X-CGD, and ADA-SCID

In this review, we will focus on the most relevant PIDs with established functional defects in NK cells, such as CNKD [5] with mutations in GATA2 [6–8], MCM4, RTEL1, GINS1, and IRF8 genes; FNKD, with defects in the FCGR3A gene and PIDs involving NK cells, like Wiskott–Aldrich syndrome (WAS) [9–11], Chèdiak–Higashi syndrome (CHS) [12, 13], Griscelli syndrome type II (GS2) [14, 15], familial hemophagocytic lymphohistiocytosis 2 (FHL2) [16, 17] and 4 (FHL4) [18–20], APDS and APDS-2 [21], severe combined immunodeficiency due to ADA deficiency (ADA-SCID), X-linked severe combined immunodeficiency (X-SCID), and X-linked chronic granulomatous disease (X-GCD) as described in detail in Fig. 1 and Table 1 [5].

**Table 1:** Overview of the most relevant PIDs, their OMIM number, their affected gene, the locus of the mutations, and the mutations themselves

Disease	OMIM number	Mutated gene	Locus	Mutation
CNKD1	614172	*GATA2*	3q21.3	c.1061C>T
IMD54	609981	*MCM4*	8q11.21	71-2A-G
DKCB	608833	*RTEL1*	20q13.33	c.3791G>A
IMD55	610608	*GINS1*	20p11.21	c.247C-T
IMD32A-32B	601565	*IRF8*	16q24.1	T80A
IMD20	615707	*FCGR3A*	1q23.3	c.230 T-A
FHL2	267700	*PRF1*	9q21.3-q22	c.1122G>A
FHL4	603552	*STX11*	6q24.2	c.173T>C
GS2	607624	*RAB27A*	15q21.3	c.582T>G
WAS	301000	*WAS*	Xp11.23-p11.22	c.3416>A
CHS	214500	*LYST*	1q42.1-q42.2	c.1902dupA
APDS	615513	*PIK3CD*	1p36.22	c.3061G>A
APDS-2	616005	*PIK3R1*	5q13.1	c.1425+1G>C,T
X-SCID	300400	*IL2RG*	Xq13.1	K97X
ADA-SCID	102700	*ADA*	20q13.12	R156H
X-CGD	306400	*CYBB*	Xp21.1-p11.4	P415H

The pathogenesis and processes underlying target recognition by human NK cells of all the PIDs mentioned before are still not well understood. For this reason, the use of induced pluripotent stem cells (iPSCs), as a disease modeling tool, will help the study of human NK cell disorders with the final goal of elucidating the disease mechanisms to find novel gene and cell therapies for the treatment of those PIDs.

## iPSC generation as a disease modeling tool

Prof. Yamanaka in 2006 made the pivotal finding that somatic cells can be reprogrammed to pluripotent cells by introducing a set of transcription factors, named induced pluripotent stem cells (iPSCs) [22]. This novel work used fibroblasts from adult mouse tail tips in order to create cells that resembled mouse embryonic stem cells morphologically and in gene expression. Essentially, these iPSCs could be proliferated and differentiated in vitro and in vivo, giving rise to mature cell types from the embryonic germ layers, endoderm, mesoderm, and ectoderm as well as to germ cells. One year later, a parallel procedure was successfully used to generate iPSCs from human fibroblasts. This achievement permitted the use of this technology in the in vitro study of human diseases [23]. The most commonly used cell type to generate iPSCs is the fibroblast, though other cell types have also been used to derive iPSCs (keratinocytes, epithelial cells, blood cells, etc.) [24]. Later, several groups reported the success of generating mature and fully differentiated blood cells, including NK cells [25].

Substantial improvement has also been made in the development of other strategies to accomplish reprogramming. Generation of iPSCs was initially based on using four retroviral vectors [26]. Recently, non-integrative methods (episomal vectors and Sendai virus, among others) have been developed to reprogram in a safer manner [27, 28]. In 2011, Eguizabal and colleagues were able to reprogram mature cells, such as dermal fibroblasts and blood cells, into iPSCs and later differentiate them into any desired cell type [25, 29–31]. To date, several studies have been reported using this technology to generate patient-derived iPSCs for disease modeling and for future applications in cell and gene therapies [32–35].

One of the first reports of a PID patient-derived iPSC line was from a patient with an adenosine deaminase (ADA) deficit, which causes severe combined immunodeficiency (ADA-SCID) [36]. While no follow-up studies using the ADA-SCID iPSCs have been reported to date, this crucial publication presented a proof of principle that iPSCs can be generated from patients with PIDs for investigating and correcting this disease. In 2011, Pessach and colleagues also published the successful generation of iPSCs from PID patients caused by specific mutations [37]. Another example of patient-specific iPSC generation was performed with cells from Fanconi anemia patients. Izpisua Belmonte’s group conducted this study, and interestingly, iPSCs could only be reprogrammed after correction of their mutation and gave rise to phenotypically normal myeloid and erythroid progenitors [38]. In addition, two papers from Malech’s laboratory show the generation of iPSCs using peripheral hematopoietic stem cells from five different genotypes of chronic granulomatous disease (CGD) patients. Both the patient iPSCs and patient somatic cells before being reprogramed into iPSCs can be corrected. Then, from the corrected iPSCs by in vitro myeloid differentiation, normal granulocytes were generated [39, 40]. In 2016, Laskowski and colleagues generated iPSCs from a Wiskott–Aldrich syndrome (WAS) patient and the WAS locus was targeted in order to produce corrected WAS-iPSCs. This group proved that the defects showed by WAS-iPSC-derived lymphoid cells were completely corrected for potential therapeutic use [41].

**Fig. 2:**
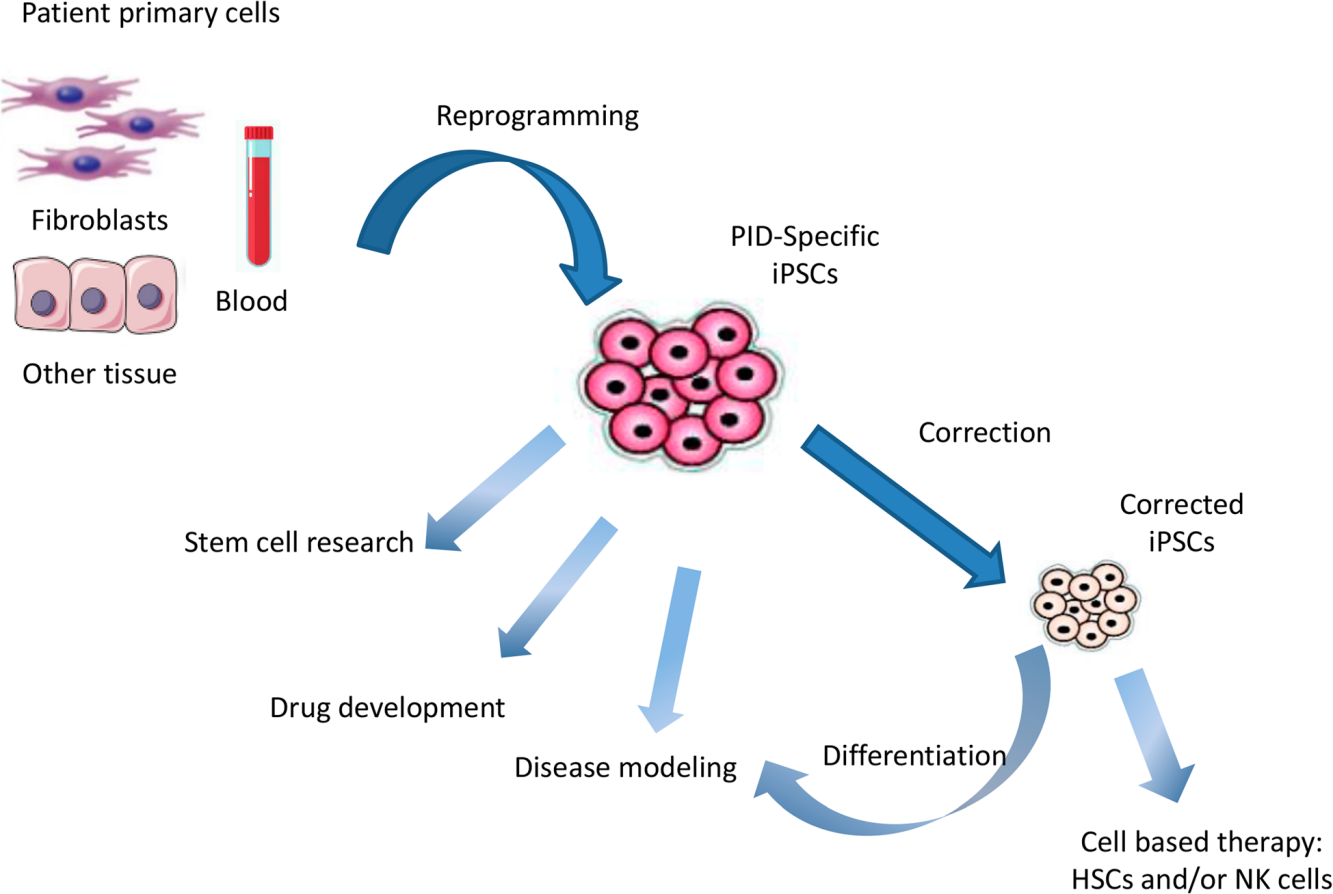
Obtaining hiPS cells from different cell sources in order to use them as a disease model, drug developmental model, or stem cell research. hiPS cells from a PID patient may be corrected with the goal of developing a cell-based therapy. Adapted from [37]

Further studies with PID-mutation-corrected iPSCs are needed to determine whether they are capable of differentiating into any target cell type as well as to gain more thorough knowledge of the mechanisms behind specific mutations [42]. Altogether, these publications reveal that disease–patient-specific iPSCs are an exceptional tool for improved understanding of human diseases and to develop novel and disease-specific cell and gene treatment approaches (Fig. 2).

## Gene editing revolution

During the DNA replication process, after experimental manipulation by using endonucleases or after exposure to chemotherapy or ionizing radiation, DNA damage can occur. Luckily, DNA repair mechanisms are able to repair this damage, thus avoiding DNA mutations that can develop into disease. This repair process is the basis of gene editing therapies, which follow two main repair pathways for double-strand breaks (DSBs) in the DNA: non-homologous end joining (NHEJ) and homologous recombination (HR) [43].

In the 1980s, pioneering scientists Prof. Capecchi, Smithies, and Evens discovered the HR pathway that repaired genes in mammalian cells. Later in 2007, they were awarded the Nobel Prize in Medicine for their findings in introducing gene modifications in mice models by using embryonic stem cells and HR-mediated gene editing. A few years later, Prof. Jasin enhanced gene targeting in mammalian cells using HR from yeast endonuclease I-SceI by using meganucleases. Since that time, next-generation gene editing tools have been used such as zinc-finger nucleases (ZFNs) and transcription activator-like effector nucleases (TALENs), which all edit DNA in combination with the FokI endonuclease [43].

In the late 1980s, a bizarre topology at the 3′ end of the alkaline phosphatase gene was revealed in *E. coli*. This was a clustered regularly interspaced short palindromic repeats (CRISPR) array, which is now one of the most commonly used gene editing technologies. Later, in 2005, the molecular mechanism was revealed, which showed that CRISPR arrays are transcribed into RNA to cleave and load into CRISPR-associated (Cas) proteins (Cas9) [43].

For many years, scientists have been looking for a tool to induce or repair mutations in a targeted manner. Several techniques have been used in the past, such as engineered meganucleases, ZFNs, and TALENs, all with limited success, due to the fact that they are labor-intensive, expensive, or both. CRISPR-based methodologies together with RNA-guided nuclease activity meant that, theoretically, DSBs in eukaryotes can be induced, which was very hard to achieve before. In general, DSBs are repaired by DNA repair pathways, with NHEJ having the potential to induce indels—mutations caused by random insertion or deletion of nucleotides at the DSB site, whereas the HR repair pathway is more precise [44].

**Fig. 3:**
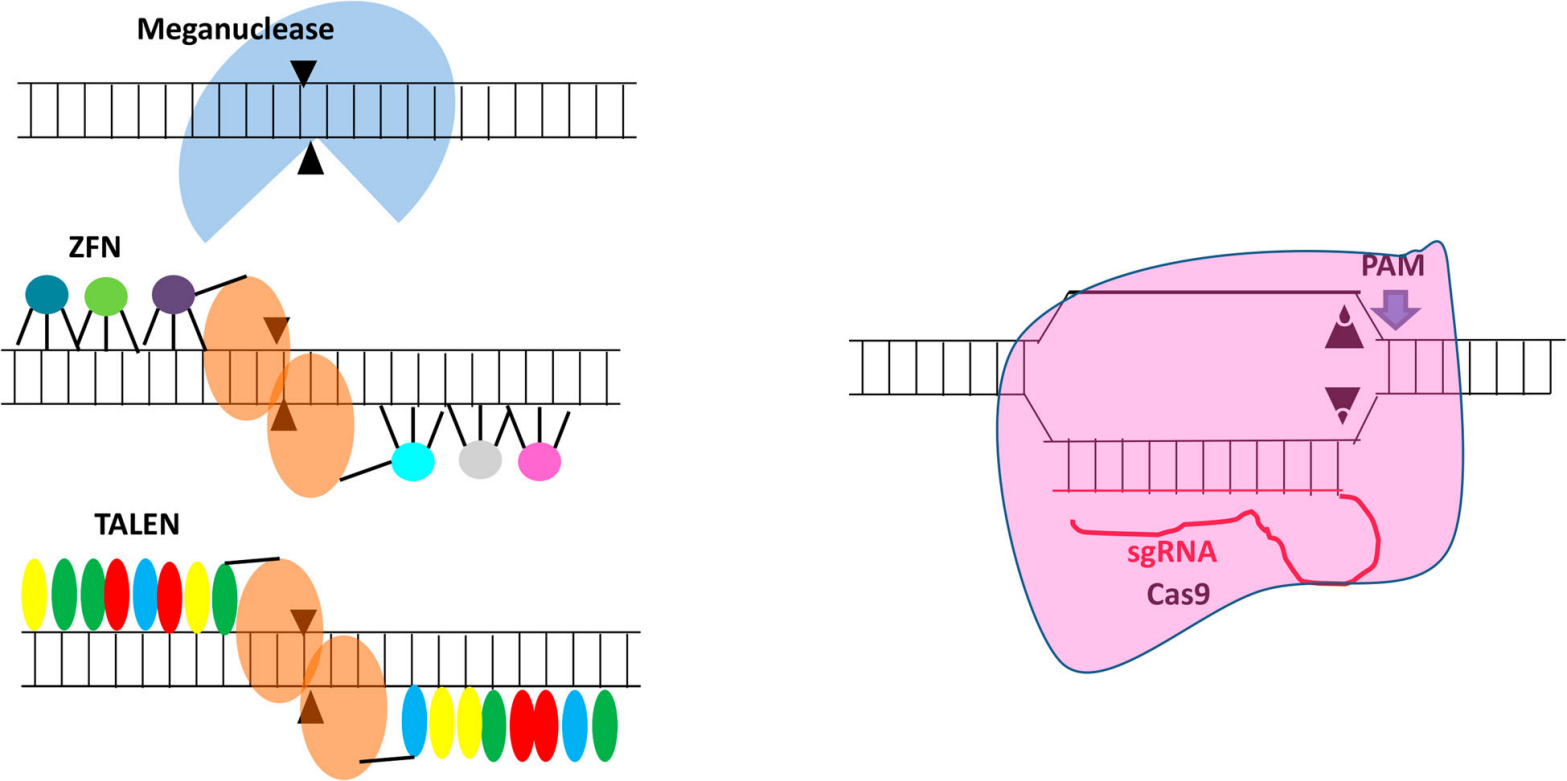
Diagram of the different types of existing gene editing tools: engineered meganucleases, zinc-finger nucleases (ZFNs), TAL effector nucleases (TALENs), and CRISPR/Cas9 nucleases

The differences between engineered meganucleases, ZFNs, TALENs, and CRISPR/Cas9 nucleases are described in detail in Fig. 3.

*Engineered meganucleases* are derived from a huge family of natural homing endonucleases [45], and some have been designed with diverse strategies (structure-based design and yeast surface display) to identify natural target sites in the genome [46, 47]. Historically, natural meganucleases have been the gold standard for specificity, but have not been fully evaluated for translational development.

*Zinc-finger nucleases* (ZFNs) are artificial restriction enzymes in which a DNA-cleavage domain from the enzyme FokI is fused to a zinc-finger DNA-binding domain [48, 49]. The nuclease domain must dimerize to cut DNA efficiently. Consequently, a pair of ZFNs oriented correctly to allow dimerization must be engineered for each target site. A variety of strategies can be engineered for novel target sites for zinc-finger DNA-binding domains (modular assembly, phage display, bacteria-based two-hybrid and one-hybrid systems, and combinatorial approaches) [50]. Though ZFN design strategies are constantly being enhanced, engineering of these recombinant proteins with high activity and specificity still remains a challenge. However, the highest-quality ZFNs generated are a mixture of phage and modular display that are in an engineered T cell clinical trial [51].

*TAL effector nucleases* (TALENs) are artificial proteins with a similar structure to ZFNs with the fusion of the enzyme FokI nuclease domain to an engineered DNA-binding domain. This DNA-binding domain is engineered by gathering serial TAL repeats [52]. Each repeat mediates the interaction with a single nucleotide through a two amino acid repeat variable di-residue (RVD) that can be described by a simple code [53]. Thus, generating a highly active TALEN is easier than generating a highly active ZFN. Moreover, TAL repeats that use engineered RVDs and not natural ones are now being used to build TALENs and may have increased specificity over natural RVDs, though this still necessitates further study. A pair of TALENs must be engineered to recognize target sites of interest, as with ZFNs; thus, TALENs using TAL repeats with RVDs have superior specificity when compared to ZFNs.

*CRISPR/Cas9 nucleases* (CRISPR stands for “clustered regularly interspaced short palindromic repeats”) originate from the immune system of bacteria and archea [54]. The specificity of the CRISPR/Cas9 nuclease system is based on RNA–DNA Watson–Crick base pairing instead of protein–DNA interaction. In this system, a single-guide RNA (sgRNA) is constructed for the 20 nucleotides matching the target region. This target site must be next to a proto-spacer adjacent motif (PAM) sequence, which the Cas9 protein uses to recognize target sites [55]. The Cas9 protein, together with the sgRNA, is capable of unwinding double-stranded DNA, cross-examine if the single-guide adequately matches the target site, and generate a double-strand break in order to repair or introduce mutations. CRISPR/Cas9 nucleases can be engineered very simply since they are active at the desired target site.

Gene editing technology is a powerful tool currently being used in basic research, but the ultimate aim is to translate these tools to be applied in therapeutic treatments. Being able to use gene editing technology in the clinic stems from the possibility of treating monogenic diseases by developing a novel method to correct the disease-associated mutation [56, 57]. There are several companies (Cellectis, Sangamo Therapeutics, Editas Company, CRISPR Therapeutics, Caribou Biosciences, Precision Biosciences, and Intellia Therapeutics) developing gene editing-based approaches to treat monogenic diseases like β-thalassemia, sickle cell anemia, cystic fibrosis (CF), hemophilia, Duchenne muscular dystrophy (DMD), alpha1-antitrypsin deficiency (A1ATD), Huntington’s disease, lysosomal storage disorders (LSDs), among others [44]. Unfortunately, no gene editing-based strategies to treat PIDs have been developed yet, but surely, they are coming soon.

Certainly, the use of gene editing tools in patient-specific iPS cells will aid in the development of future treatments aimed at correcting the point mutations in PIDs with defects in NK cells.

## Current gene and cell therapies for PIDs with defects in NK cells

**Fig. 4:**
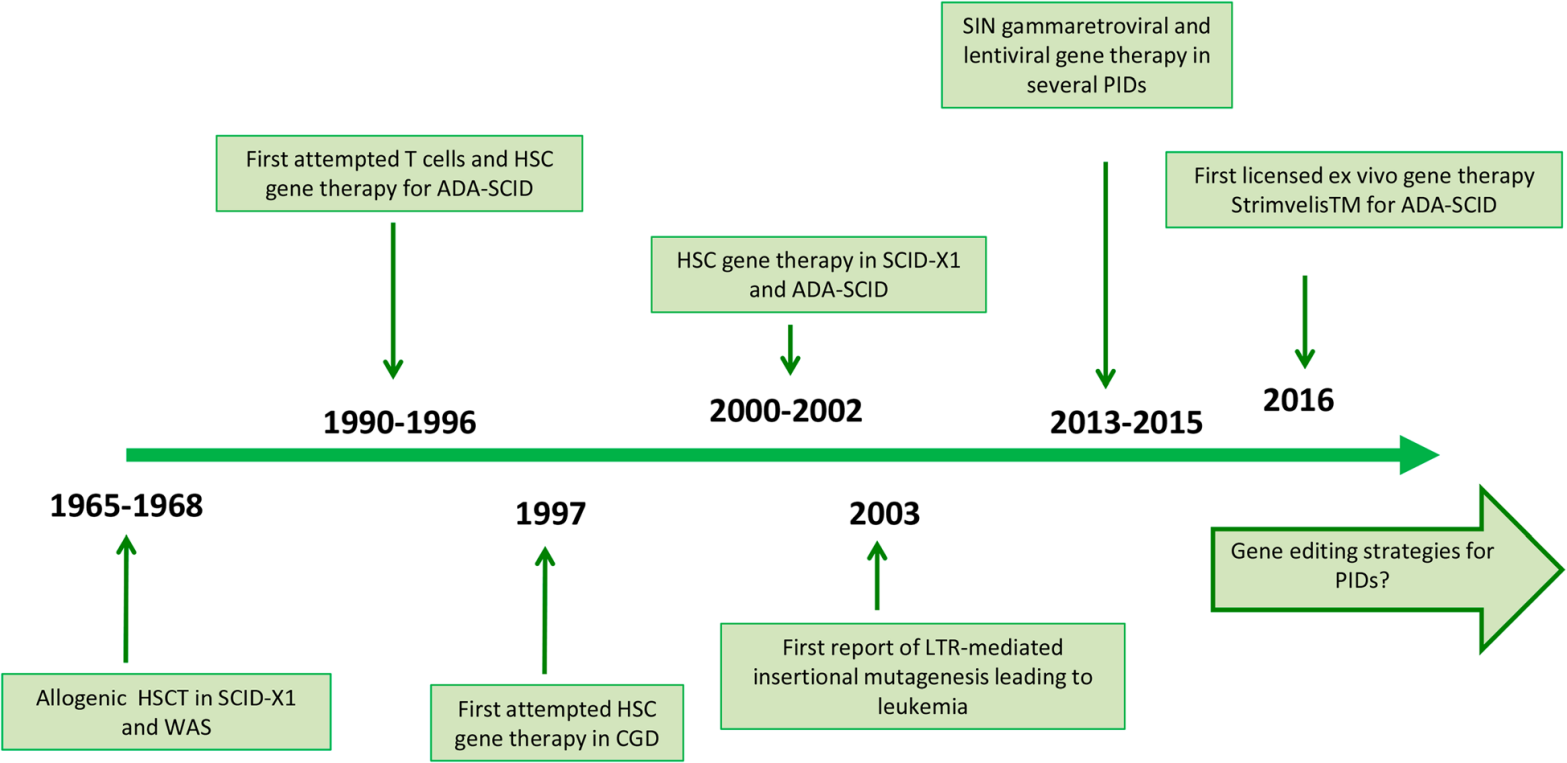
Viral vector technology development and its application to human gene therapy. The line represents the timeline of this technology, from the 1960s to now. Adapted from [59]

The first time HSCT was used as a therapeutic option for treating PID in a severe combined immunodeficiency (SCID) patient was in 1965 [58], as shown in Fig. 4**.** Since then, HSCT has been the standard care for SCID patients with great survival rates [60–62]. However, depending on the HLA-match, HSCT could have some complications. On the one hand, when an HLA-identical sibling donor is available, the success rates are very high. On the other hand, a less well-watched HLA allogenic donor, such as haploidentical family members or unrelated donors, could lead to some serious risks. Among these risks, the more worrisome ones are graft rejection and graft versus host disease (GvHD). If either of these conditions occur, patients require an urgent restoration of hematopoiesis to prevent complications from prolonged pancytopenia [63]. In order to avoid these risks, during the last 20 years, genetic manipulation of a patient’s own autologous immune cells has been studied and is being translated into clinical trials. Key clinical trials using g-retroviral vectors for gene correction in adenosine deaminase SCID, Wiskott–Aldrich syndrome, and X-linked SCID had moderate hematopoietic cell transduction efficiency. Nevertheless, these trials served as a proof of concept that gene correction could provide a curative therapy since treated patients showed improvement in immune function. Unfortunately, several years after treatment, patients developed leukemia as a consequence of the activation of pro-oncogenes close to the g-retroviral vector insertion points [64]. As a consequence, this led to the development of lentiviral vectors, which improved the efficacy and safety of the gene therapies. For example, Strimvelis (gene therapy for ADA-SCID) is now open in Europe [65, 66] (Fig. 4). Currently, there are very few clinical trials that combine cell and gene therapy that are ongoing for several PIDs as shown in Table 2 [67, 68, 69], but none utilizes gene editing strategies.

**Table 2:** Overview of current available clinical trials for different PIDs

Disease	Mutated gene/protein	Vector/target cell	Conditioning	Clinical trial reference
CNKD1	*GATA2*	Allogeneic HSCT	Busulfan/fludarabine/cyclophosphamide/TBI	NCT01861106
WAS	*WAS*	SIN-LV/BM/PBSCs	RIC busulfan/fludarabine	NCT01515462
				NCT01347346
				NCT01347242
				NCT01410825
		SIN-LV/PBSCs	None	NCT03837483
X-SCID	*IL2RG*	SIN-γRV/BM	None	NCT01410019
				NCT01129544
				NCT01175239
		SIN-LV/PBSCs	Busulfan 6 mg/kg	NCT01306019
		SIN-LV/BM	Busulfan 6 mg/kg	NCT01512888
ADA-SCID	*ADA*	SIN-LV/BM/PBSCs	Busulfan 5 mg/kg	NCT01380990
		SIN-LV/BM/PBSCs	Busulfan 4 mg/kg	NCT01852071
				NCT02022696

*SIN-γRV* self-inactivating γRV vector, *SIN-LV* self-inactivating lentiviral vector, *BM* bone marrow, *PBSCs* peripheral blood stem cells, *MAC* myeloablative conditioning, *RIC* reduced intensity conditioning, *TBI* total body irradiation

The triumph of gene therapy in treating PIDs is a major advancement, though limitations in manufacturing disease-specific vectors remain a challenge [70]. As this field moves forward, more efficient procedures offering wider spread applications arise. Gene editing defines a group of DNA editing approaches that can be simply designed for point mutations. Recently, programmable nucleases such as ZFNs, TALENs, and CRISPR-Cas9 have been developed as effective methods for editing the genome to correct the affected gene in PIDs [49, 71–76].

Compared to lentiviral vectors, gene-specific editing technologies has become a tremendously promising tool, as it has the potential to physiologically regulate gene expression and prevent genome-wide vector integration. Some of the ongoing efforts are focused on developing sensitive techniques to detect genotoxicity derived from unintended effects of endonucleases (off-target effects).

In the case of CRISPR-Cas9 approaches for HSC genome editing in PIDs, the design of the donor template is challenging and both the nature (single/multiple mutations or deletions in one or more hotspots distributed along the gene) and the functional effect of the mutation (gain of function versus loss of function) have to be taken into consideration [77].

Short donor templates (such as ssODN or linear or plasmid dsDNA donors) have been used to correct loss of function (LOF) mutations of a single or few nucleotides. For example, De Ravin and colleagues [78] could repair the mutation in the CYBB gene of CD34^+^ HSCs from patients with the immunodeficiency disorder X-linked chronic granulomatous disease (X-CGD) using a chemically modified 100-bp ssODN that resulted in production of 15–20% functional mature human myeloid and lymphoid cells for up to 5 months.

In contrast to small mutations, repair of large deletions or insertions is not possible with short donor templates and instead functional complementary DNA (cDNA) templates are inserted to target genes. Encouraging preclinical studies have been published using this approach for the treatment of X-SCID or X-CGD [79–81] and will be ready to translate to clinical trials soon.

However, one limitation to consider for the application of gene editing tools in a clinical setting might be the engraftment efficiency and HSC functionality of genetically modified cells due to cellular effects of the gene editing machinery. Indeed, global gene expression changes have been observed upon delivery of CRISPR-Cas9 machinery components into the cells. Immune response to viral infection, DNA damage response, apoptosis, and cell cycle processes have been reported as the most significantly enriched gene signatures [82]. The activation of these biological processes might negatively affect HSC stemness and hematopoietic lineage expansion and differentiation. Further studies are needed in order to better understand these mechanisms and therefore design more efficient CRISPR-Cas9 strategies and improve HSC engraftment efficiency.

Apart from CRISPR-Cas9, other genome editing tools have been used to modify genes in different cell types including HSCs. ZFN and TALEN techniques have been used to modify the IL2RG locus, which is responsible for SCID [83].

Specifically in the case of PID with NK cell defects, a good example of the evolution of treatment approaches is seen with WAS. The first clinical trial with gene therapy in WAS patients was performed using gamma-retroviral vectors. Even if 9 of 10 patients showed partial or complete resolution of immunodeficiency, autoimmunity, and other malignancies, 7 of them developed acute leukemia. This study demonstrated that gene therapy for WAS can be effective, although it was essential to find an alternative to gamma-retroviruses given the high risk of leukemia after months or years [84]. More recently, self-inactivating lentiviral vectors have shown efficacy for several PIDs, including WAS, and they are now in phase I/II clinical trials for a number of immune disorders. To date, more than 20 patients have been treated using lentiviral vectors and no evidence of vector-related toxicity has been observed with any reports of leukemia [85]. However, platelet recovery has been variable in those trials [86]. Although no preclinical studies have been published yet, nuclease-based gene editing approaches for repairing mutations in PID with NK cell defects might represent the future of gene therapy, already demonstrated by studies targeting other PIDs, as explained above. Hence, WAS gene targeting systems have been already tested in cell lines, providing the first hints for feasibility of CRISPR-based and heterodimeric ZNF-based gene therapy strategies [87].

In summary, thanks to a better understanding of stem cell biology, bone marrow transplantation, vector design, and genome editing, it is probable that gene therapy will become the gold standard of care for certain diseases in the future. In fact, its benefits have already been demonstrated for WAS, ADA, SCID, and X-CGD. In addition, a number of preclinical studies using targeted gene editing strategies show promise [59, 79–81, 88] and a large number of patients treated so far in clinical trials indicate that the gene therapy field is fast becoming a therapeutic standard.

## Conclusions and future perspectives

During recent years, the genome editing field, together with cell reprogramming field, has shown tremendous progress. Currently, the first clinical trials with iPSC-derived NK cells (FT500) and gene-edited somatic cells have started. Here, we described the advantages of modeling and correcting PIDs with gene editing technologies while avoiding the use of viral vectors. However, further refinement of the genome editing tools is necessary if they are to be used in a clinical setting for PID treatment. Specifically, off-target mutagenesis has to be examined and the yield of gene-corrected HSCs or other blood cells (NK cells) needs to be improved so that an adequate number of cells for autologous transplantation and engraftment can be achieved. In spite of these issues, the influence of genome editing and stem cell therapies on modern medicine will be revolutionary for the PID field.

## Data Availability

Not applicable

## Abbreviations

PIDs: Primary immunodeficiency diseases
NK: Natural killer
CTLs: Cytotoxic T lymphocytes
CNKD: Classical NK deficiencies
FNKD: Functional NK deficiencies
WAS: Wiskott–Aldrich syndrome
CHS: Chèdiak–Higashi syndrome
GS2: Griscelli syndrome type II
FHL2/ FHL4: Familial hemophagocytic lymphohistiocytosis 2 and 4
APDS: Activated PI3K-delta syndrome
PASLI: p110δ-activating mutation causing senescent T cells, lymphadenopathy, and immunodeficiency
DC: Dendritic cells
WASP: WAS protein
HLH: Hemophagocytic lymphohistiocytosis
MTOC: Microtubule-organizing center
EBV: Epstein–Barr virus
PI3K: Phosphoinositide 3-kinase
CMV: Cytomegalovirus
GOF: Gain of function
HSCT: Hematopoietic stem cell transplantation
XLP-1: X-linked lymphoproliferative syndrome type 1
iPSCs: Induced pluripotent stem cells
ADA: Adenosine deaminase
SCID: Severe combined immunodeficiency
CGD: Chronic granulomatous disease
DSB: DNA double-strand break
NHEJ: Non-homologous end joining
HR: Homologous recombination
ZFN: Zinc-finger nuclease
TAL: Transcription activator-like
TALEN: Transcription activator-like effector nucleases
CRISPR: Clustered regularly interspaced short palindromic repeats
RVD: Repeat variable di-residue
sgRNA: Single-guide RNA
PAM: Proto-spacer adjacent motif
CF: Cystic fibrosis
DMD: Duchenne muscular dystrophy
A1ATD: Alpha1-antitrypsin deficiency
LSD: Lysosomal storage disorder
HIV-1: Human immunodeficiency virus-1
HBV: Hepatitis B virus
CAR T: Chimeric antigen receptor T cell
X-CGD: X-linked chronic granulomatous disease
