# Correction to: Treating primary immunodeficiencies with defects in NK cells: from stem cell therapy to gene editing

**DOI:** 10.1186/s13287-021-02281-1

**Published:** 2021-04-27

**Authors:** C. Eguizabal, L. Herrera, M. Inglés-Ferrándiz, J. C. Izpisua Belmonte

**Affiliations:** 1 Cell Therapy, Stem Cells and Tissues Group, Biocruces Bizkaia Health Research Institute, Barakaldo, Spain; 2 Research Unit, Basque Center for Blood Transfusion and Human Tissues, Osakidetza, Galdakao, Spain; 3 Gene Expression Laboratory, The Salk Institute for Biological Studies, 10010 North Torrey Pines Road, La Jolla, California 93027 USA

**Correction to: Stem Cell Res Ther (2020) 11:453**

**https://doi.org/10.1186/s13287-020-01964-5**

After publication of our article [1], the authors became aware that they’d omitted acknowledgement of permission to adapt and reproduce Fig. 2 (first published in Current Opinion in Allergy and Clinical Immunology, 2011 [2]) and Fig. 4 (first published in Molecular Therapy, 2017 [3]).

**Fig. 2:**
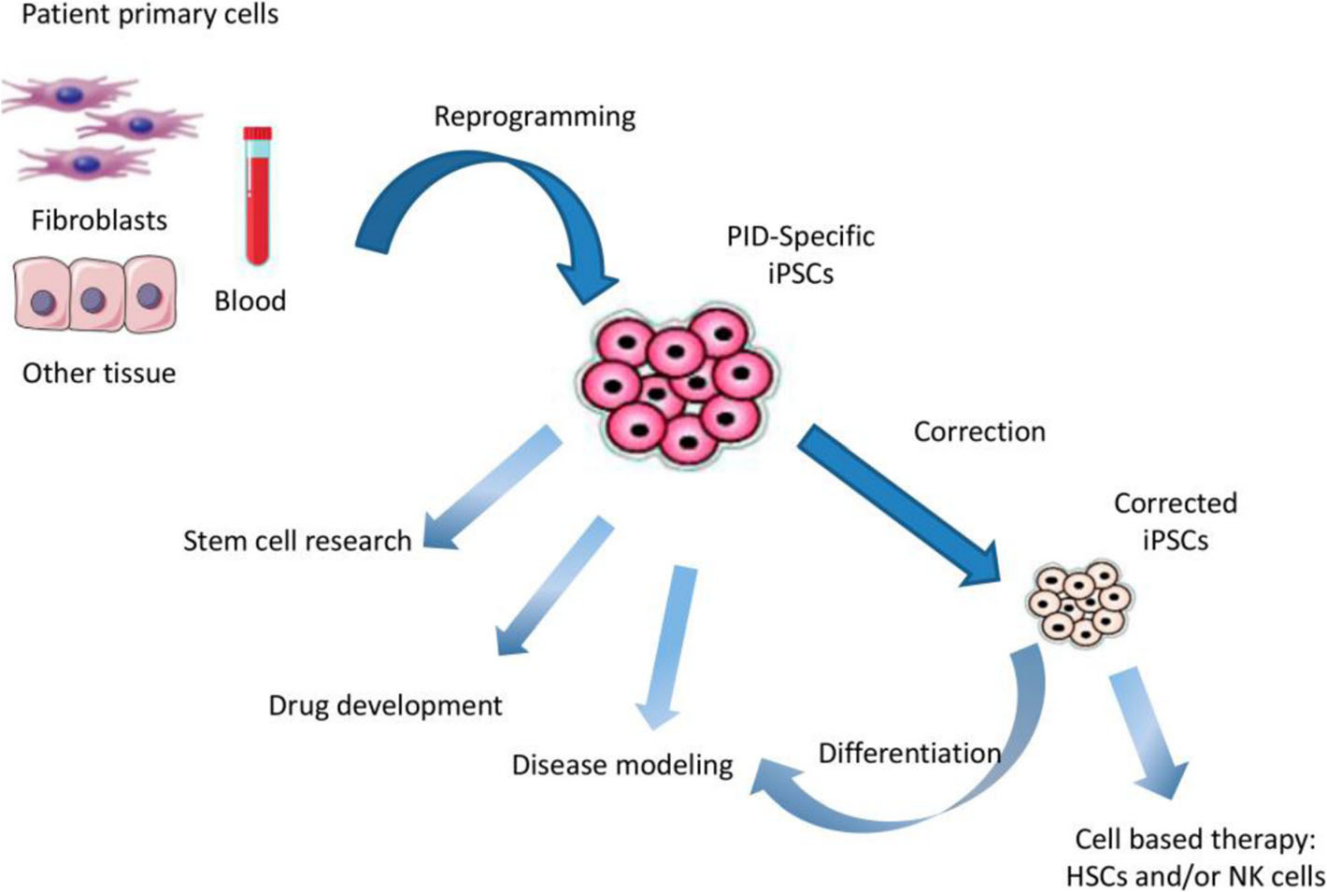
Obtaining hiPS cells from different cell sources in order to use them as a disease model, drug developmental model, or stem cells research. hiPS cells from a PID patient may be corrected with the goal of developing a cell based therapy. Adapted from [37]

**Fig. 4:**
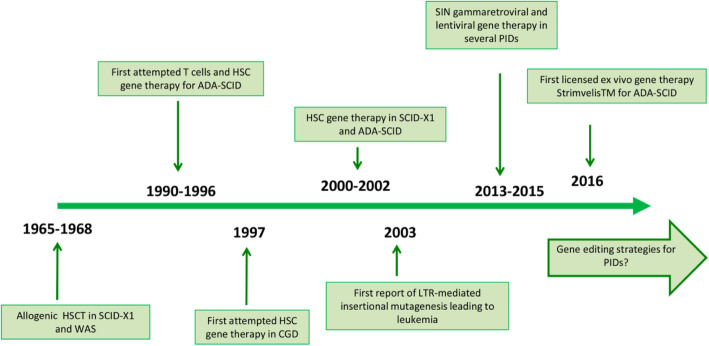
Viral vector technology development and its application to human gene therapy. The line represents the timeline of this technology, from the 1960s to now. Adapted from [59]

Figure 2 was adapted and published with permission of the Publisher. Original source: Pessach IM and Notarangelo LD. Primary immunodeficiency modeling with induced pluripotent stem cells. Curr Opin Allergy Clin Immunol. 2011 Dec;11(6):505-11. © 2011 Wolters Kluwer Health, Inc. https://journals.lww.com/co-allergy/pages/default.aspx All rights reserved. The Creative Commons license does not apply to this content. Use of the material in any format is prohibited without written permission from the publisher, Wolters Kluwer Health, Inc. Please contact permissions@lww.com for further information.

Figure 4 was adapted and published with permission of the Publisher. Original source: Thrasher AJ and Williams DA. Evolving Gene Therapy in Primary Immunodeficiency. Mol Ther. 2017 May 3;25(5):1132-1141. © 2017 The American Society of Gene and Cell Therapy. All rights reserved.

In addition, a number of reference errors were introduced which are clarified below:

- References 59-87 in the original article (references [3–31] in this correction article) have had their information corrected and also replace the original references for the respective citations in the original article text.
- Reference 37 in the original article (reference [32] in this correction article) has had its information corrected.

Section: “**CURRENT GENE AND CELL THERAPIES FOR PIDs WITH DEFECTS IN NK CELLS”**

…

Currently, there are very few clinical trials that combine cell and gene therapy that are ongoing for several PIDs as shown in **TABLE 2** (67, 68), but none utilize gene-editing strategies.

The triumph of gene therapy in treating PIDs is a major advancement, though limitations in manufacturing disease-specific vectors remain a challenge (69). As this field moves forward, more efficient procedures offering wider spread applications arise. Gene editing defines a group of DNA editing approaches that can be simply designed for point mutations. Recently, programmable nucleases such as ZFNs, TALENs, and CRISPR-Cas9 have been developed as effective methods for editing the genome to correct the affected gene in PIDs (49, 70–75).

Compared to lentiviral vectors, gene-specific editing technologies has become a tremendously promising tool, as it has the potential to physiologically regulate gene expression and prevent genome-wide vector integration. Some of the ongoing efforts are focused on developing sensitive techniques to detect genotoxicity derived from unintended effects of endonucleases (off-target effects).

In the case of CRISPR-Cas9 approaches for HSC genome editing in PIDs, the design of the donor template is challenging and both the nature (single/multiple mutations or deletions in one or more hotspots distributed along the gene) and the functional effect of the mutation (gain of function versus loss of function) have to be taken into consideration (76).

Short donor templates (such as ssODN or linear or plasmid dsDNA donors) have been used to correct loss of function (LOF) mutations of a single or few nucleotides. For example, De Ravin and colleagues (77) could repair the mutation in the CYBB gene of CD34^+^ HSCs from patients with the immunodeficiency disorder X-linked chronic granulomatous disease (X-CGD) using a chemically modified 100 bp ssODN that resulted in production of 15-20% functional mature human myeloid and lymphoid cells for up to 5 months.

In contrast to small mutations, repair of large deletions or insertions is not possible with short donor templates and instead functional complementary DNA (cDNA) templates are inserted to target genes. Encouraging preclinical studies have been published using this approach for the treatment of X-SCID or X-CGD (78–80) and will be ready to translate to clinical trials soon.

However, one limitation to consider for the application of gene editing tools in a clinical setting might  be the engraftment efficiency and HSC functionality of genetically modified cells due to cellular effects of the gene-editing machinery. Indeed, global gene expression changes have been observed upon delivery of CRISPR-Cas9 machinery components into the cells. Immune response to viral infection, DNA-damage response, apoptosis and cell cycle processes have been reported as the most significant enriched gene signatures (81). The activation of these biological processes might negatively affect HSC stemness and hematopoietic lineage expansion and differentiation. Further studies are needed in order to better understand these mechanisms and therefore design more efficient CRISPR-Cas9 strategies and improve HSC engraftment efficiency.

Apart from CRISPR-Cas9, other genome editing tools have been used to modify genes in different cell types including HSCs. ZFN and TALEN techniques have been used to modify the IL2RG locus, which is responsible for SCID (82).

Specifically in the case of PID with NK cell defects, a good example of the evolution of treatment approaches is seen with WAS. The first clinical trial with gene therapy in WAS patients was performed using gamma-retroviral vectors. Even if 9 of 10 patients showed partial or complete resolution of immunodeficiency, autoimmunity and other malignancies, 7 of them developed acute leukemia. This study demonstrated that gene therapy for WAS can be effective, although it was essential to find an alternative to gamma-retroviruses given the high risk of leukemia after months or years (83). More recently, self-inactivating lentiviral vectors have shown efficacy for several PIDs, including WAS and they are now in Phase I/II clinical trials for a number of immune disorders. To date, more than 20 patients have been treated using lentiviral vectors and no evidence of vector-related toxicity has been observed with any reports of leukemia (84). However, platelet recovery has been variable in those trials (85). Although no pre-clinical studies have been published yet, nuclease-based gene editing approaches for repairing mutations in PID with NK cell defects might represent the future of gene therapy, already demonstrated by studies targeting other PIDs, as explained above. Hence, WAS gene targeting systems have been already tested in cell lines, providing the first hints for feasibility of CRISPR-based and heterodimeric ZNF-based gene therapy strategies (86).

In summary, thanks to a better understanding of stem cell biology, bone marrow transplantation, vector design and genome editing, it is probable that gene therapy will become the gold standard of care for certain diseases in the future. In fact, its benefits have already been demonstrated for WAS, ADA, SCID and XCGD. In addition, a number of preclinical studies using targeted gene editing strategies show promise (78–80, 86, 87) and a large number of patients treated so far in clinical trials indicate that the gene therapy field is fast becoming a therapeutic standard.
